# Dental problems and oral microbiome alterations in ulcerative colitis

**DOI:** 10.3389/fimmu.2025.1502605

**Published:** 2025-02-05

**Authors:** Robert Kucharski, Bartosz Kamil Sobocki, Ewa Stachowska, Nikola Bulman, Leszek Kalinowski, Karolina Kaźmierczak-Siedlecka

**Affiliations:** ^1^ Department of Medical Laboratory Diagnostics – Fahrenheit Biobank BBMRI.pl, Medical University of Gdansk, Gdańsk, Poland; ^2^ Neodentica Dentistry Center, Gdansk, Poland; ^3^ Department of Oncology and Radiotherapy, Medical University of Gdansk, Gdańsk, Poland; ^4^ Department of Human Nutrition and Metabolomics, Pomeranian Medical University in Szczecin, Szczecin, Poland; ^5^ BioTechMed Center, Department of Mechanics of Materials and Structures, Gdansk University of Technology, Gdansk, Poland

**Keywords:** ulcerative colitis, oral microbiome, periodontitis, dental caries, oral ulcers, lichen planus, dental care

## Abstract

Ulcerative colitis is a chronic disease that has not well-established etiology. The role of microbial dysregulation in its pathogenesis has been recently highlighted. Overall, microbiome alterations concern the reduction of bacterial abundance and diversity, resulting in gut microbiome imbalance negatively affecting immunological aspects. There is a link between ulcerative colitis and the oral microbiome. The changes of oral microbiome are found at many levels, from gently dysbiotic composition to the presence of the main periodontal microbes. The analysis of oral microbiome can be a part of personalized medicine due to the fact that it is a potential biomarker. Patients with ulcerative colitis may manifest dental symptoms/problems, such as periodontitis (strongly related to the red-complex pathogens—*Porphyromonas gingivalis*, *Tannerella forsythia*, *Treponema denticola*, and bacteria belonging to the other complexes, such as *Fusobacterium nucleatum* and *Aggregatibacter actinomycetecomitans*), dental caries, oral ulcerations, leukoplakia, halitosis, and others. Notably, the DMFT (Decayed, Missing, Filled Teeth) index is higher in these patients compared to healthy subjects. According to some data, oral lichen planus (which is a disease with an immunological background) can also be observed in ulcerative colitis patients. It seems that deep understanding of ulcerative colitis in association with oral microbiome, immunology, and dental manifestations may be crucial to provide complex treatment from a dental point of view.

## 1 Introduction

Ulcerative colitis (UC) is one of the main subtypes in the group of inflammatory bowel diseases (IBDs) (1, 2). The incidence of UC is still increasing, especially in industrialized countries (3, 4). There were 5 million patients with UC in 2023 (5). UC is a chronic disease that affects the colon, causing mucosal inflammation from distal to proximal part (finally involving the whole colon and rectum) (6–8). Although UC can develop at any age, the peak is observed from the second to fourth decade of life (3). The etiology of UC is still not fully understood; however, it affects people with genetic predisposition and environmental exposure on some factors, such as Western diet, smoking, air pollution, stress, and particular medications (3, 9–13). Currently, it is estimated that only 8% to 14% of patients suffering from UC present a family history of IBDs (3). An inappropriate, high-fat diet is related to the increased risk of UC. Fritsch et al. reported that a low-fat diet reduces both markers of inflammation and intestinal dysbiosis in UC patients in remission/mild period of the disease (14). The pathogenesis of UC is associated with microbial dysregulation. Overall insights into gut microbiome (GM) in patients with UC are related to changes in biodiversity and bacterial abundance as well as dysbiotic composition (15, 16), including a decline in butyrate-producing bacteria (such as *Roseburia* spp. and *Faecalibacterium prausnitzii*) and beneficial species (like *Bifidobacterium longum* and *Akkermansia muciniphila*). There is also evidence suggesting decreases in *Lactobacilli*, *Bacteroides fragilis*, and *Veillonella* spp., along with an increase in potentially harmful bacteria, including *Proteobacteria* and *Clostridium* spp. Sulfate-reducing bacteria like *Desulfovibrio* are also implicated due to their role in producing hydrogen sulfide, which can exacerbate mucosal damage (17, 18). Recently, Kang et al. demonstrated the possibility of diagnosing both Crohn’s disease and UC using fecal whole metagenome shotgun sequencing and a machine learning predictive model (19). Microbiome-immune-related aspects regard chronic inflammation, high level of pro-inflammatory mediators, loss of tight junctions, increase of intestinal permeability, and dysregulation of T-cell response (16). The occurrence of inflammation can be influenced by some bacteria through affecting the differentiation of T-cell subsets (20). For instance, *Bacteroides fragilis* may have an impact on the ratio Th1/Th2 by Toll-like receptor 2 (TLR2). Clostridium clusters IV, XIVa, and XVIII via TGF-β may affect the differentiation of Treg cells (18). Not only the bacterial microbiome is altered in UC but also the fungal part. Interestingly, a severe form of UC is related to the genetic polymorphisms in the fungal-sensing gene—*Dectin-1* (21). Hsia et al. have shown that in the case of UC, endoscopic inflammation amount of *Saccharomyces* and *Candida* was increased, whereas in endoscopic remission, amount of these fungi was reduced (21). Therefore, considering the level of *Saccharomyces* and *Candida* may be helpful in the context of fungal biomarkers and personalized medicine.

It should be emphasized that UC is a disease that not only affects the colon but also causes pathological changes in the oral cavity (22, 23). It is estimated that, in adults, oral manifestations of overall IBDs range from 0.7% to 37% (24). Tan et al. reported that patients with Crohn’s disease have significantly more oral health problems than UC subjects (25). Although oral manifestations in UC can be observed, it is still not a well-discussed problem. For instance, Zeng et al. presented a case of a 52-year-old male with two extra-intestinal manifestations, that is, pyostomatitis vegetans in the oral cavity as well as Sweet syndrome (on the skin) (26). Dimmock et al. also reported case of a UC patient treated with infliximab who developed pyostomatitis vegetans (27). Notably, oral health problems are associated with the impairment of IBD-specific health-related quality of life (28). Due to the fact that the link of intestinal microbiome and UC is intensively analyzed in literature, in the current paper we presented oral microbiome changes, immunological background, and the most significant dental problems in the case of UC, providing a multidisciplinary point of view.

## 2 Oral microbiome in UC

Many findings related to the relationship between the oral microbiome and UC were revealed, raising questions about the direction of this relationship (UC affecting the oral microbiome, or the oral microbiome affecting UC, or mutual influence), and highlighting potential therapeutic perspectives (29). The oral microbiome is the second part of the microbiome in terms of diversity and composition after the GM. Generally, in healthy conditions, it is usually significantly different (29–31). Certainly, UC seems to influence the composition of the oral microbiome. It was shown in the limited cohort of patients (10 UC vs. 11 healthy controls, 16S rRNA gene sequencing) that UC might cause oral dysbiosis with decreased alpha diversity and the increased abundance of, for example, *Proteobacteria*, as well as *Neisseriaceae*, and the decreased levels of *Peptostreptococcaceae* and *Lachnospiraceae* compared to healthy subjects. Interestingly, exclusively Staphylococcus members and four differential species/phenotypes were found in the UC patient’s saliva (32). These findings were also investigated in another similar study (10 UC patients vs. 10 healthy subjects, 16S rRNA gene-based analysis). Although significantly reduced alpha diversity in UC patients was not confirmed (only a tendency, *p* = 0.06), the results of the study have shown oral microbiome misbalance, including, for instance, increased abundance of *Proteobacteria*, *Actinobacteria*, and *Candidate Division* TM7 while decreased *Bacteroidetes* and *Verrucomicrobia* in UC patients. Moreover, the increase of *Streptococcus*, *Staphylococcus*, and *Veilonella* levels was observed (33). The study by Said et al. indicated higher abundance of *Prevotella* and *Veilonella* and lower *Streptococcus* and *Haemophilus* levels in saliva samples from UC patients compared to healthy subjects (respectively 14 vs. 24 subjects). Moreover, it was revealed that lysozyme level was lower whereas immunological biomarkers (IL-1β, IL-6, IL-8, MCP-1) were higher in the UC group (34). One of the biggest studies conducted by Xun et al. compared the salivary bacterial DNA of 54 UC patients versus 25 healthy individuals (using the 16S rRNA V3-V4 region) and showed enrichment of *Streptococcus* and *Veilonella* as well as depletion of *Prevotella* and *Neisseria* in the UC group. In addition, this dysbiosis was correlated with impaired white blood cell levels, reduced basic metabolic processes, and stimulation of oxidative stress and virulence (via excreted substances) (35). Another study investigated the impact of periodontal salivary microbiota on gut inflammation and the induction of UC in the experimental model of the disease (salivary microbiota was collected from healthy individuals and those with periodontitis and gavaged to C57BL/6 mice) (36). The measurements with 16S rRNA gene sequencing, flow cytometry, and liquid chromatography-mass techniques (as the main techniques used in the study) showed that the presence of salivary periodontitis-related microbiota worsened dextran sulfate sodium-induced colitis. Moreover, it stimulated aggravated M2 macrophage polarization and Th2 induction. In addition, the periodontitis-related oral microbiome stimulated colonic inflammation, the decrease of unsaturated fatty acid levels, and the increase of arachidonic acid metabolism. The study underlined changes in levels of *Aerococcus*, *Ruminococcus*, *Blatia*, and *Helicobacter* in experimental colitis (36). This was consistent with more general observations by Schirmer et al. (study in 405 pediatric UC patients) in which it was proven that the disease severity correlated with depletion of typical gut microbes and expansion of bacteria typical to the oral cavity (37). On the other hand, a study that enrolled 31 pediatric patients with UC and 43 healthy subjects showed no significant differences in microbial diversity in samples from buccal and tongue mucosal brushings between the cohorts (38). Furthermore, the interesting study by Xu et al. showed that oral microbiome anatomical distribution may have functional implications. Although the study compared small populations (10 UC patients having oral ulcers vs. 22 UC patients without them, 16S rRNA V3-V4 region sequencing), it showed that patients with oral ulcers had weaker treatment responses, and probably *Fusobacterium*, *Oribacterium*, and *Campylobacter* played a major role in that matter (39). Moreover, the study by Szczeklik et al. (13 UC patients vs. 3 controls) indicated that the total oxidative potential of OM was higher in patients with UC than in healthy subjects. However, the study should be interpreted carefully because of the limited cohort (40). Some studies might suggest oral microbiome as a factor leading to gut changes, but the strength of proof is really weak (41, 42).

This interrelation of oral microbiome and GM has been already described (43–45). It was proven that in UC the composition of the GM and oral microbiome was significantly more similar than in healthy subject samples (45). Potential mechanisms of UC pathogenesis induced by dysbiosis, among others, include impaired activation of the immune system caused by translocation of pathogenic bacteria to the gut (e.g., *Staphylococcus*), short-chain fatty acid deficiency (e.g., by *Lachnospiraceae* level reduction), the impairment of epithelial barrier function, and toxin production (46–48). Nevertheless, the mechanism of action in this case should be further investigated and validated. It is not certain what is first: oral or GM dysbiosis. Some pathogens present in periodontal disease (e.g., *Fusobacterium nucleatum*, *Klebsiella*, *Porphyromonas gingivalis*, *Helicobacter pylori*, *Streptococcus*, *Veilonella*, *Parvimonas micra*) reach the gut via swallowed saliva, and it is a certain way of transmission leading to gut dysbiosis (41, 49). On the other hand, some studies proved that induced mice models of UC subsequently change oral microbiome composition, indicating that oral microbiome change is not always before UC (50). There is a need for new, prospective, international research studies in human subjects with numerous cohorts of UC patients, focusing on the molecular interrelation of both oral microbiome and GM that address still valid questions about the origin and mechanisms of UC development.

There are also some projects focusing on improving the diagnosis. One of them looked for potential oral microbial biomarkers to differentiate UC from Crohn’s disease (CD) and healthy subjects (16S rRNA sequencing data from saliva samples, UC: *n* = 175, CD: *n* = 124, healthy subjects: *n* = 99). The public datasets Kraken2 and SILVA were used to train the model. The alpha diversity was significantly lower in UC and CD than in healthy subjects. The created model sPLS-DA was able to differentiate both diseases from each other (accuracy around 85) and HS (accuracy around 90) (19). Also, another model in pediatric patients was created, based on a limited number of subjects (11 with UC and 8 HS) with accuracy above 85% using oral and fecal samples (51). However, these models are still quite complex and may not be easy to introduce into clinical practice. More representative, broadly characterized, prospectively obtained, real-world data are needed to enable the development of more advanced models reflecting a diversified population (19).

## 3 Therapeutic methods of microbiome modification

### 3.1 Probiotics

A potential way of affecting the disease is probiotics. Systematic review and network meta-analysis based on 42 studies, 839 UC models and 24 different probiotics showed their huge impact on UC outcomes, including the reduction of colon injury, disease activity index, and serum pro-inflammatory factor TNF-alpha, as well as the increase in the expression of tight junction proteins and overall improvement in autoinflammatory reaction, mucosal barrier regeneration, and the histopathological manifestations caused by some probiotics (52). Study of Heavey et al. revealed that *Saccharomyces boulardii* might be a good candidate in this context (53). In a mice model, they created a yeast platform where this probiotic was designed to specifically bind to abundant inflamed regions of gut extracellular matrix proteins. It leads to increased therapeutic concentration and subsequent improved colon length, cytokine expression profile, and histological inflammation score, which, after treatment, were comparable with healthy conditions. This bacteria was able to stimulate resident microbes to produce SCFAs as well as modulate local gut immune response (to anti-inflammatory phenotype, e.g., by inhibition of the NFκB pathway) (53). SCFAs are known for reducing immune response and increasing intestinal barrier integrity (54). Hao et al. in a mice model showed the potential of *Lactobacillus plantarum* Q7-derived vesicles. This probiotic increased the microbiota diversity and anti-inflammatory bacteria (*Bifidobacteria* and *Muribaculaceae*) while decreasing pro-inflammatory *Proteobacteria* and the levels of proinflammatory cytokines. Altogether it alleviated DSS-induced UC (55). Another study that investigated *Lactobacillus plantarum* HNU082 confirmed its beneficial role. In the DSS-induced mice model, the symptoms were alleviated after supplementation and the mechanism of action was dependent on the decrease of pro-inflammatory cytokines and the increase of anti-inflammatory ones (IL-10, transforming growth factor-β1 TGF-β1, and TGF-β2), as well as on improving the mechanical barrier by increasing Zonula occludens proteins and claudins (1 and 2) (56). Very similar conclusions and design were also applied in a study investigating *Veilonella ratti* and *Lactobacillus acidophilus.* The combination of these two had the same effect on cytokines and further stimulated the production of SCFAs and anti-inflammatory enzymes (SOD and GSH) (57). In the same mice model, Li et al. showed a beneficial effect of *Lycium Barbarum* polysacchardies, also inhibiting proinflammatory cytokines, increasing anti-inflammatory IL-10, and tight junction protein levels (Occluding and ZO-2) as well as improving the abundance of *Ruminococcaceae*, *Lactobacillus*, *Butyricicoccus*, and *Akkermansia* (58). In addition, Jia et al. showed that *Lactobacillus johnsonii* improved DSS-induced mice model through activation of native macrophages into CD206+ type, and TLR1/2-STAT-mediated release of IL-10 (59). Regulation of macrophages on the way to silence UC-related inflammation was also shown by Sun et al., who pointed to the role of engineered *Saccharomyces cerevisiae* that suppressed macrophage pyroptosis, parallelly producing beneficial lactic acid inhibiting the production of proinflammatory cytokines (60). Guo et al. formed *Lactobacillus casei* into a pericellular film, which provided a polysaccharide network for spatially restricted crystallization of selenium dots. This study showed that the shape matters, as in multiple mouse models and non-human primates, the colonic damage and inflammation were reduced more in pericellular film than in classical probiotic form (61). On the other hand, it is important to apply probiotics in careful way, as some cases of sepsis due to them were noted (62). The clinical trials showed promising results, considering that probiotics reduced the risk of clinical relapse, increased the chance for clinical remission, decreased inflammatory markers (e.g., calprotectin), and improved quality of life (63, 64). Considering the research mentioned above, there is a need not only to test new probiotics in clinical setting but also to investigate new methods of delivery and implementation of bacteria in the gut. The problem to face is also the sustainability of oral and GM changes. A study of 21 subjects clearly showed that after supplementation of *Lactobacilli* and *Streptococci*, the composition of the oral microbiome increased in diversity, but the structure of the microbiome was not significantly changed, and the effect was short term (65). Unfortunately, the studies with positive outcomes in oral microbiome long-term changes were not revealed. Given the changes in GM after successful therapy, they will not be persistent if oral microbiome is still impaired. Therefore, there is a need to develop new solutions related to binding probiotics also in the oral cavity to increase their abundance there.

### 3.2 FMT

Another promising option is fecal microbiota transplantation (FMT). A double-blind, randomized, placebo-controlled trial (15 patients received FMT vs. 20 placebo patients) showed that antibiotics followed by lyophilized oral FMT resulted in remission of UC in the majority of patients. Three patients were affected by serious adverse events: two patients experienced worsened UC (two in the FMT group, one in the placebo group), and one per-rectal bleeding (in the placebo group). Ten patients after clinical or endoscopic confirmation of response (FMT group) were randomly distributed to groups who continued FMT (*n* = 4) and discontinued (*n* = 6) the treatment. All patients in the first group achieved stable clinical, endoscopic, and histologic remission at week 56, whereas patients who discontinued therapy experienced progression of disease (66). Another clinical trial in adult patients (*n* = 73) showed that patients after FMT had a higher likelihood of remission at 8 weeks (67). One clinical trial found that multidonor FMT might be even more effective (*n* = 66) in combination with an anti-inflammatory diet, as it caused deep remission (36.4%), remission (60%), and overall clinical response (65.7%) in a significant population of patients that lasted over 1 year and surprisingly was superior to standard medical therapy (68). The double-blind clinical trial of 81 patients revealed that higher abundance of *Fusobacterium gonidiaformans*, *Sutterella wadsworthensis*, and *Escherichia* species and increased levels of heme and lipopolysaccharide biosynthesis were related to resistance to FMT (69). Therefore, its eradication or replacement by probiotics prior to treatment seems to be crucial. A systematic review and meta-analysis of congregated available literature support these results (70–73).

Collected papers seem to support hypotheses about the involvement of the oral microbiome and periodontal disease in the pathogenesis of UC by, for instance, the influence on GM and negative impact on immune cell composition or intestinal tight junctions. However, the current status of knowledge provides only a partial explanation of the mechanism. The variety of potential probiotics was listed in this chapter, indicating that they influence mainly anti-inflammatory and pro-inflammatory signaling pathways and strengthen the tight junction proteins function and their abundance. An interesting combination might be probiotics with FMT, which has already been clinically validated as a treatment strategy. Hopefully, some of them may contribute to reducing the UC burden. The future of UC treatment might be the combination of anti-inflammatory drugs with these modulating the oral and GM. Further research needs to address these questions.

## 4 Dental problems in UC

### 4.1 Periodontitis

Periodontitis is a chronic disease that occurs as a result of coexisting factors, such as microbial pathogens, environmental factors (for instance, smoking), and susceptible hosts (74). It is reported that periodontitis is the sixth most common human disease (75). Periodontitis leads to multiple consequences. One of the major local problems is tooth loss. The chronic inflammation in periodontitis is strongly associated with red-complex bacteria regarding *Porphyromonas gingivalis*, *Tannerella forsythia*, and *Treponema denticola*. Other bacteria—*Fusobacterium nucleatum* and *Aggregatibacter actinomycetecomitans—*are also deeply related to periodontitis (76, 77). *P. gingivalis* (anaerobic, Gram-negative bacterium) is known as a major periodontal pathogen with several virulence factors (gingipains, capsule, fimbriae, outer membrane proteins) by which it stimulates, among others, inflammation (74, 78). As it was mentioned above, *F. nucleatum* (anaerobic oral bacterium) participates in the development of periodontitis (79). Interestingly, according to some data, the amount of *F. nucleatum* is increased in the stool of patients with IBD (80). The mechanisms by which *F. nucleatum* causes intestinal inflammation is complex. One of them regards upregulation of cytokine secretion (for instance, pro-inflammatory IL-6) and activation of the STAT3 signaling pathway. Recently, in 2023, it was shown that *F. nucleatum* promotes UC by contributing to gut microbiota dysbiosis, epithelial barrier dysfunction, as well as dysmetabolism (49). Currently it is also suggested that the FadA gene is important in the pathogenesis of UC, due to the fact that in these patients the presence of *F. nucleatum* and FadA gene were increased. Nevertheless, the mechanism is still not well discovered (79). The pathological background of the link between UC and oral problems (regarding also periodontitis) potentially regards both oral and intestinal dysbiosis (imbalance), dysregulation of the immune system, as well as malnutrition (81). Negative changes of the immune system include alterations of periodontitis-related factors, that is, reduction of anti-inflammatory IL-10 and increase of TNF-α and MMP-8 (matrix metalloproteinase 8). Malnutrition is caused by malabsorption from inflamed intestines, changes of digestion as well as absorption due to using drugs, and low food intake (81). Poor dietary habits promote gut microbial imbalance, which can consequently contribute to the development of UC. Notably, periodontitis is associated with the translocation of bacteria to the digestive system (75). As it was mentioned above, Qian et al. analyzed the impact of periodontitis salivary microbiota on inflammation in the colon (36). This study was conducted with dextran sulfate sodium (DSS)-induced colitis C57BL/6 mice receiving salivary microbiota from healthy subjects or periodontitis. It was observed that microorganisms, such as *Blautia*, *Helicobacter*, and *Ruminococcus*, were altered in DSS-induced colitis gavaged with periodontitis salivary microbiota. The authors concluded that periodontitis is involved in the pathogenesis of colitis (36). Enver et al. assessed periodontal status as well as levels of cytokines in both saliva and gingival crevicular fluid in the case of patients with IBDs (82). This study included 131 patients divided into three groups: UC, Crohn’s disease, and non-IBD. It was noted that patients with UC diagnosed with periodontitis have higher scores of bleeding on probing (*p* = 0.011) and increased level of pro-inflammatory IL-1β compared to subjects with Crohn’s disease. Moreover, this cytokine was associated with the parameters representing the severity of periodontal diseases in the active form of Crohn’s disease and UC (82). In another study it was shown that more patients with IBD demonstrate moderate/severe periodontitis (85.6% vs. 65.6%, *p* < 0.001) or severe form of this disorder (36.7% vs. 25.6%, *p* < 0.001) compared to healthy controls (75). Bertl et al. in a questionnaire-based case-control study (regarding 1,108 IBD patients and 3,429 controls) presented that patients suffering from IBD declared worse oral health and more periodontal problems compared to the healthy subjects (83). Interestingly, Tan et al. analyzed DMFT (Decayed, Missing, Filled Teeth) scores and DPSI (Dutch Periodontal Screening Index) of IBD patients (84). Considering all IBD groups, it was observed that the total DMFT index was significantly higher in this group compared to control subjects. However, this observation was also confirmed in patients with Crohn’s disease, whereas not in UC patients when the analysis was conducted separately. Based on DPSI, there was no significant difference between IBD and non-IBD groups (84). The prevalence of periodontitis and DMFT index were also analyzed in Brito et al. study (83). It was reported that significantly more patients with UC (90%, *p* < 0.001) and Crohn’s disease (81.8%, *p* = 0.03) had periodontitis compared to control subjects. Additionally, smoking was related to the periodontitis in UC (85). Smoking has a negative effect on both the incidence and progression of periodontitis (86). According to the new classification scheme for periodontal and peri-implant diseases and conditions, smoking is one of the risk factors, which allows to assess the grade of periodontitis (grade A: slow rate of progression, B: moderate, C: rapid) (87).

### 4.2 Dental caries

Dental caries is one of the most common oral diseases globally. Notably, around 2.3 billion people have untreated caries in permanent teeth (88). As it is mentioned above, the DMFT index is higher in IBD patients than in healthy individuals. Zhang et al. also noted that IBD patients present a higher prevalence of dental problems (regarding dental caries/periodontal disease) compared to the control subjects (89). A similar observation was found in a systematic review and meta-analysis (90). Factors such as eating sweets and inefficient dental plaque removal contribute to the development of dental caries. Rodrigues et al. have shown that the DMFT index in UC patients was not affected by the frequency of eating foods containing sugar between meals (91). However, higher prevalence of dental caries was associated with long term disease (*p* = 0.06). Moreover, the amount of cariogenic bacterium *Mutans streptococci* was increased in patients with active form of UC and longer duration (91). The mechanisms/causes by which dental caries are more commonly detected in UC are not yet well understood. It can be related to dry mouth (which is one of the oral problems in UC patients), alterations of salivary and microbiological aspects (24). Another reason can be associated with the fact that these patients are paying more attention to underlying disease, that is, UC, and low levels of oral hygiene, dietary habits (consuming a refined carbohydrate diet), and lack of dentist visits.

### 4.3 Oral ulcerations

Oral ulcers, which are one of the most common signs in the oral cavity in UC, cause pain and discomfort (92). According to the data provided by Singhal et al., patients with IBDs had a higher frequency of oral ulcers (*p* = 0.04) and other oral problems (dental caries *p* = 0.007, dry mouth *p* = 0.001) compared to healthy subjects (92). General prevalence of oral lesions in IBD patients is 5%–50% (93). Sun et al. presented a case of a 52-year-old male with UC and aphthous ulceration and oral epithelial dysplasia (93). Oral changes were effectively treated using triamcinolone acetonide oral ointment and mouthwash (lidocaine, gentamicin, dexamethasone), resulting in healing after one week of therapy (93). The response to the treatment in UC patients with oral ulcers can be negatively modulated by three oral bacterial genera, such as Fusobacterium, Oribacterium, and Campylobacter, which has been recently shown in Xu et al. study (39). In these patients, aphthous ulcerations can also be detected. They may be caused by several factors, such as intestinal malabsorption and rectal bleeding, which lead to the deficiency of vitamins (B_12_) as well as minerals (iron, zinc) (24).

### 4.4 Oral lichen planus

Lichen planus is known as a chronic inflammatory disease, which can also affect the oral mucosa, causing oral variant of lichen planus (94). It is estimated that 1%–2% of the population developed oral lichen planus (95). It can also be detected in UC patients (96). There are six types of oral lichen planus, that is, reticular, plaque-like, papular, atrophic/erosive, ulcerative, and bullous (95). It is often located in the buccal mucosa, tongue, as well as gingiva. The pathogenesis of oral lichen planus regards antigen-specific mechanisms [(1) antigen presentation by keratinocytes and Langerhans cells to CD4+ and CD8+ T lymphocytes; (2) the release of pro-inflammatory mediators IL-2 and INF-γ; (3) activation of cytotoxic T lymphocytes, apoptosis of basal keratinocytes, and degeneration of basal epithelial cells] and non-specific [(1) degranulation of mast cells, release of TNF-α as well as chymase; (2) the activation of matrix metalloproteinase-9 by chymase; (3) destruction of basement membrane and migration of CD8+ cells into the epithelium of lesions; (4) apoptosis of basal and parabasal epithelial cells] (97–99). Currently, it is also known that oral lichenoid drug reactions are associated with both mesalazine and sulfasalazine (medications used in IBD treatment) (24).

## 5 Cases of oral alterations in UC from dental practice

**Figure 1:**
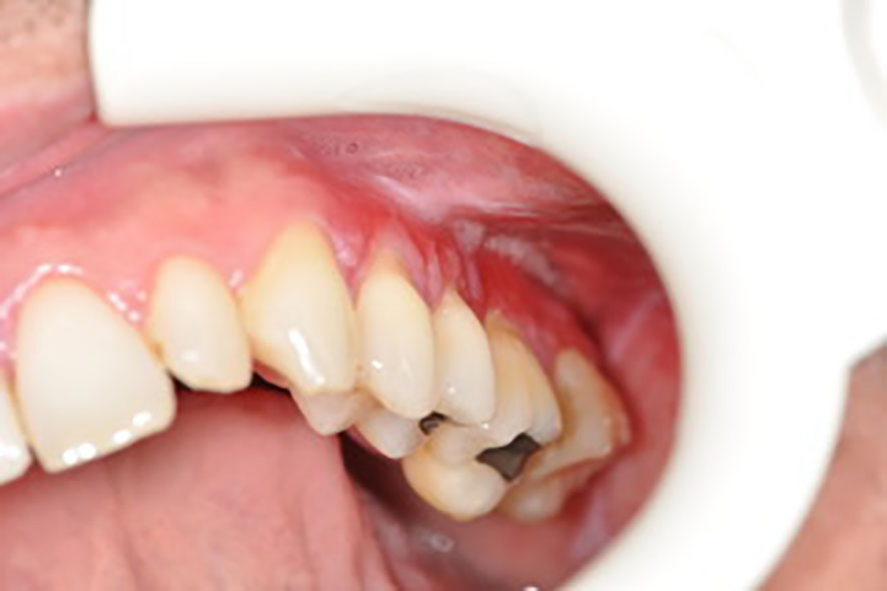
Leukoplakia.

**Figure 2:**
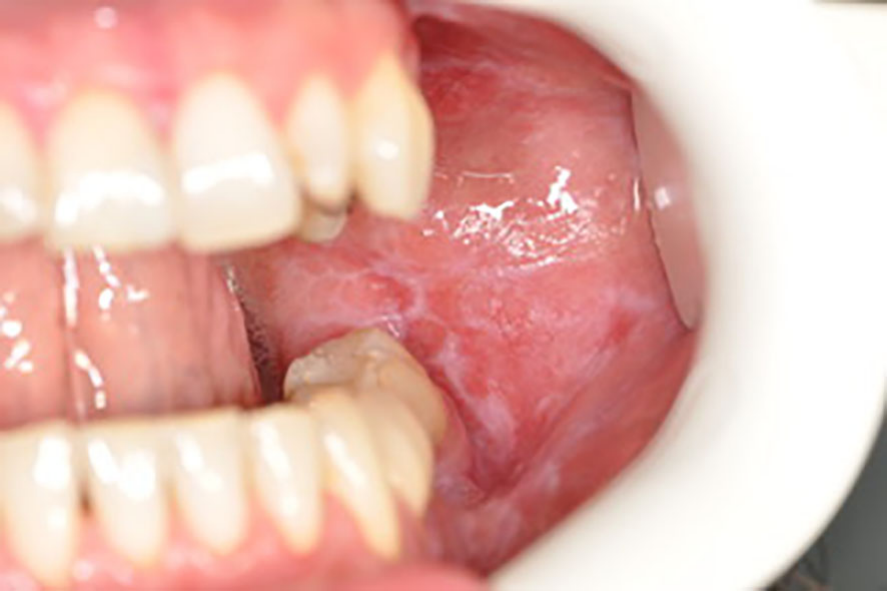
Wilson’s lichen.

**Figure 3:**
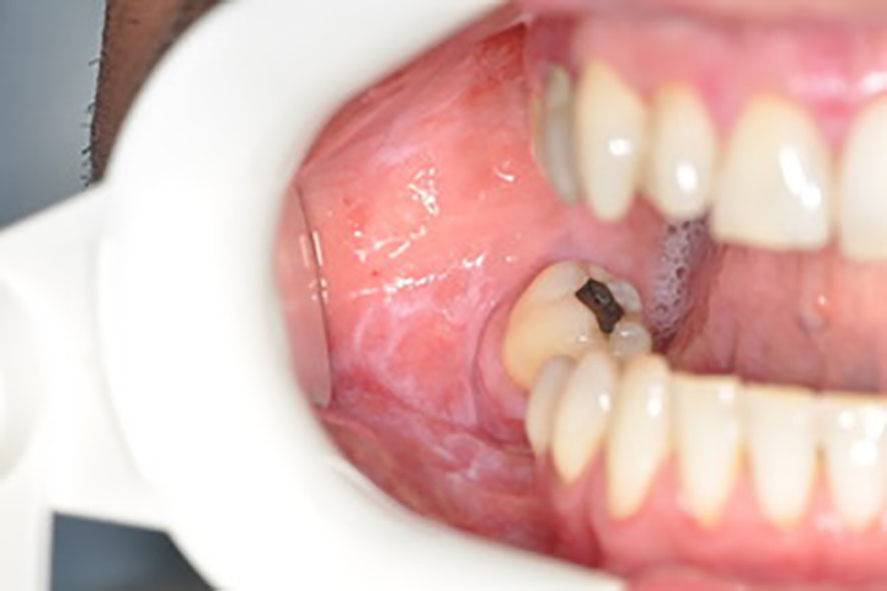
Wilson’s lichen.

Below there are presented the pathological changes in oral cavity in case of UC observed in dental practice (**Figures 1**–**3**).

## 6 Dental care in UC

Routine dental care in case of IBD is similar to that of the general population. However, according to the above-discussed studies, dentists should pay more attention to the possible problems that may occur in the oral cavity. Singhal et al. reported that IBD patients more frequently visited dentists at disease onset (*p* < 0.001) compared to the healthy controls (92). It could be associated with several factors. IBD patients are treated with steroids and immunosuppressive medications, which increase the incidence of oral symptoms (*p* = 0.052) and the risk of infections (99, 100). It should be taken into consideration, especially in the case of complicated oral dental procedures, for instance, in the case of surgical treatment. According to the best practice, it is recommended to create an individual plan of treatment for UC patients. For instance, in the case of immunosuppressive patients, “one shot” of antibiotic (amoxicillin 2 g or clindamycin 600 mg) is given one hour prior to the introduction of surgical treatment if the procedure lasts less than an hour (in another case, i.e., >1 hour, the full antibiotic introduction should be provided—amoxicillin for 7 days or clindamycin for 5 days). It is also significant to pay more attention to the level of vitamin D_3_ due to its strong connection with the immune system. However, the individual plan should be introduced (101). Additionally, the active period of this disease is related to the development of more dental/oral problems. For instance, Katz et al. observed that the active period of UC was associated with a higher prevalence of halitosis (*p* < 0.001) (102). To sum up, special attention from dentists is strongly needed in the case of these patients.

## 7 Conclusions

Oral pathologies, which are observed in UC patients, can be caused by several factors, such as medications (corticosteroids, immunomodulators, antibiotics), malnutrition, vitamin deficiencies (A, C, D, B_12_, and others), microbiome dysbiotic alterations, inappropriate oral hygiene, and lifestyle (for instance, smoking). They are developed with different frequency among patients with UC. It should be noted, especially in the case of oral changes, which are rarely found, to avoid overlooking and to provide appropriate dental care. Oral manifestations may additionally negatively affect patients’ quality of life. It confirms the strong need for multidisciplinary and complex treatment of UC. The standard management regards gastroenterologists and surgeons; however, the role of dentists is also important to avoid additional complications and improve quality of life.
